# The Impact of the Ubiquitin System in the Pathogenesis of Squamous Cell Carcinomas

**DOI:** 10.3390/cancers12061595

**Published:** 2020-06-16

**Authors:** Veronica Gatti, Francesca Bernassola, Claudio Talora, Gerry Melino, Angelo Peschiaroli

**Affiliations:** 1 National Research Council of Italy, Institute of Translational Pharmacology, 00133 Rome, Italy; veronica_gatti@yahoo.it; 2 Department of Experimental Medicine, TOR, University of Rome Tor Vergata, 00133 Rome, Italy; bernasso@uniroma2.it (F.B.); melino@uniroma2.it (G.M.); 3 Department of Molecular Medicine, Sapienza University of Rome, 00185 Rome, Italy; claudio.talora@uniroma1.it

**Keywords:** squamous cell carcinoma, E3 ubiquitin ligase, deubiquitinating enzymes, oncogenes, tumor suppressor

## Abstract

The ubiquitin system is a dynamic regulatory pathway controlling the activity, subcellular localization and stability of a myriad of cellular proteins, which in turn affects cellular homeostasis through the regulation of a variety of signaling cascades. Aberrant activity of key components of the ubiquitin system has been functionally linked with numerous human diseases including the initiation and progression of human tumors. In this review, we will contextualize the importance of the two main components of the ubiquitin system, the E3 ubiquitin ligases (E3s) and deubiquitinating enzymes (DUBs), in the etiology of squamous cell carcinomas (SCCs). We will discuss the signaling pathways regulated by these enzymes, emphasizing the genetic and molecular determinants underlying their deregulation in SCCs.

## 1. Introduction

### 1.1. Molecular Landscape of SCCs

Squamous cell carcinomas (SCCs) are highly common and malignant solid cancers that arise from stratified and pseudo-stratified epithelia of the skin, and the aerodigestive and genitourinary tracts. Several environmental carcinogenic insults contribute to the development and pathogenesis of SCCs. UVA and UVB exposure is a major risk factor for cutaneous SCCs while cigarette smoking is linked to lung (LSCCs), esophageal (ESCCs) and head and neck (HNSCCs) SCCs. The etiology of HNSCCs is also strictly associated with infections with Epstein–Barr and human papilloma (HPV) viruses [1]. SCCs are hard to treat, and patients have poor overall 5-year survival rates, which is about 50% for HNSCCs, 18% for LSCCs and 66% for cervical SCCs (CvSCCs) [2,3,4]. The standard therapeutic approach includes surgery, combined with radiotherapy and chemotherapy. Few targeted molecular therapies include epidermal growth factor (EGF-R) receptor inhibitor (cetuximab) for HNSCCs [5,6] and antibody to VEGF (bevacizumab) for CvSCC [7]. Recently, immune checkpoint inhibitors (pembrolizumab and nivolumab) are being used for the treatment of HNSCCs, LSCCs and CvSCCs [6,7,8,9].

Genome sequencing and mRNA/miRNA expression studies have allowed identifying a molecular and genetic signature shared by different SCC types, which distinguish SCCs from other tumors. Interestingly, HPV-positive cancers are the less heterogeneous types, which show lower DNA copy number alteration coupled with higher CpG methylation levels and lower mRNA expression [1,10]. Mutational analysis of cancer-related genes showed that the most frequently mutated gene in different SCCs is the tumor suppressor p53 [11,12,13,14,15]. The mutational rate of *TP53* varies between different SCCs: 61–95% in CSCC, 47–100% in HPV negative HNSCC, 82–93% in ESCCs and 72–91% in LSCCs [1]. Missense “hot spot’’ mutation lead to loss- or gain-of-function features promoting survival, invasiveness and drug resistance [16,17,18,19,20,21]. Other relevant genetic hits commonly found in SCCs affect key determinants of the cell cycle machinery (CDKN2A deletion, c-Myc or cyclin D1 amplification), pro-survival and mitogenic signals (PIK3CA mutations, EGF-R and FGF-R amplification), component of the epigenetic system (FAT1, KMT2D and KMT2c mutations) and the squamous cell differentiation program (*TP63, NOTCH, IRF6, SOX2* and *ZNF750*) [10,11,12,13,14,22].

### 1.2. Overview of the Ubiquitin System

**Figure 1:**
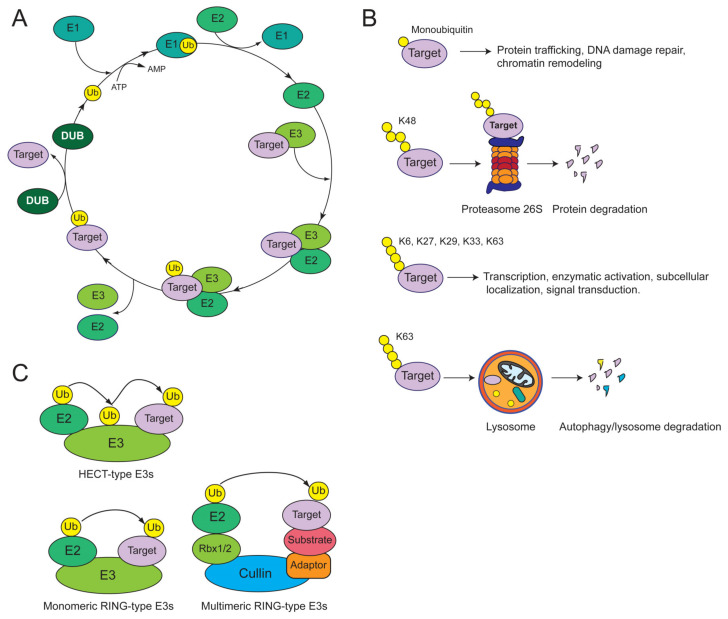
(**A**) The addition of ubiquitin to a target protein is a multistep, ATP-dependent process performed in three sequential enzymatic steps: activation of ubiquitin by an E1 ubiquitin activating enzyme, transfer of ubiquitin to one E2 ubiquitin conjugating enzyme and, lastly, the E3 ligase-mediated transfer of ubiquitin from E2 to the substrate protein. Ubiquitination can be reversed by deubiquitinating enzymes (DUBs), a family of isopeptidases that catalyze the removal of ubiquitin from target proteins. (**B**) Fate of ubiquitin-modified proteins. (**C**) Structural architecture and catalytic properties of HECT- and the RING finger-type E3s. HECT-type E3s form a thioester-linked intermediate with ubiquitin before catalyzing its transfer to the substrate. The RING-type E3s act as platforms for recruiting the E2 thioester-linked to ubiquitin and for juxtaposing the E2 and the substrate for the ubiquitin transfer. This family comprises of both monomeric and the multi-subunit E3s.

Multiple evidence indicated that SCCs development is also functionally linked with dysregulation of the ubiquitin system, an intracellular protein modification pathway that catalyzes the reversible covalent attachment of ubiquitin to Lys residues on target substrates [23]. Protein ubiquitination occurs through the sequential and coordinate action of three biochemical activities: an E1 ubiquitin activating enzyme (E1), an E2 ubiquitin conjugating enzyme (E2) and an E3 ubiquitin protein ligase (E3; Figure 1A). The ubiquitination cascade initiates with the adenylation of the ubiquitin C-terminal carboxyl group (termed ubiquitin “activation”) by the E1, which, subsequently, catalyzes ubiquitin transfer to a Cys active site residue on itself. The thioester-linked ubiquitin is then transferred to a specific Cys residue of an E2, via a similar thioester linkage. E3 ligases (E3s) interact with both the ubiquitin-loaded E2 and the substrate protein. The E3s are responsible for substrate recognition and for promoting the transfer of ubiquitin to substrate proteins. Proteins can be modified by monoubiquitination on a single Lys residue, multi-mono ubiquitination as a result of the attachment of a single ubiquitin to multiple sites, or polyubiquitination as result of the sequential ubiquitin attachment to the same Lys residue. Notably, ubiquitination can also occur at the first methionine.

Different types of polyubiquitin chains can be generated by using one of the seven Lys residues of the acceptor ubiquitin molecule (K6, K11, K27, K29, K33, K48 and K63) or, alternatively, the N-terminal methionine residue (M1) [24,25,26]. A chain can be formed by one linkage type (homotypic ubiquitin chain) or can contain several linkage types (heterotypic ubiquitin chain) in mixed or branched polymers [27]. The fate of ubiquitin-modified proteins is determined by the type of ubiquitination (mono-, multi-mono- or poly-ubiquitination), the length and linkage type in a polyubiquitin chain as well as by other topological aspects (homotypic vs. heterotypic or mixed vs. branched ubiquitin chains; Figure 1B). Monoubiquitination preferentially facilitates protein recognition, subcellular localization and allosteric regulation. K48-linked polyubiquitin chains and, at a lesser extent K29 and K11 polymers, are the signals triggering the recognition and proteolysis of protein substrates by the proteasome [28]. Substrates conjugated to K63-linked ubiquitin chains as well as monoubiquitinated proteins are preferentially degraded by the autophagy/lysosome system [29]. Interestingly, the degradation of many cellular proteins by 26S proteasome or autophagy is triggered by ubiquitination occurring at the N-terminal residues, the so-called N-degron pathways [30].

Since E3s regulate both substrate specificity and the efficiency of ubiquitin transfer, they are crucial components of the ubiquitin system, and as such, their dysregulation leads to several human disorders, including cancer development [31,32,33,34]. On the basis of their functional domains, catalytic properties and mechanism of action, the E3s have been classified into two major groups: the homologous to E6-AP C-terminal (HECT) domain- and the really interesting new gene (RING) domain-containing E3s (Figure 1C). The HECT-type E3s comprises monomeric proteins that possess intrinsic catalytic activity, while the RING-type E3s are characterized by the presence of a RING catalytic domain, which engages and positions the E2 to promote the ubiquitin transfer to a substrate protein [35]. The RING-type E3s include both monomeric enzymes and the multi-subunit Cullin-RING ubiquitin ligases (CRLs), with over two hundred known members in humans [36,37]. CRLs share similar structural architecture and are classified in seven classes (CRL1-CRL7) on the basis of the type of cullin subunit (Cul1, Cul2, Cul3, Cul4A, Cul4B, Cul5 and Cul7) forming the complex. The prototype of the CRL complexes is the SCF-complex, which is composed of four core components: the Cul 1 protein subunit, the E2-interacting RING protein Rbx1/2, the adaptor protein Skp1 and one of the 68 different F-box proteins that act as substrate receptors by recognizing specific target proteins. CRLs activity is mainly regulated through cycles of cullin neddylation (modification by the ubiquitin-like protein Nedd8) and de-neddylation. When cullins are neddylated, they are competent to induce productive E2 engagement with Rbx1/Roc1 [38,39,40,41].

The action of E3s is counteracted by the activity of the deubiquitinating enzymes (deubiquitinases; hereafter DUBs), specialized proteases that remove ubiquitin modifications, hydrolyzing isopeptide linkages between ubiquitin molecules or between ubiquitin and the target proteins [42]. The human genome codifies for approximately a hundred DUBs, which can be classified into distinct groups based on the structural motifs of the catalytic domain. Similar to the ubiquitination process, the nature of ubiquitin chain modifications catalyzed by the DUBs controls many aspects of protein physiology such as protein stability, activity and intracellular localization [43]. 

In the next paragraphs, we will describe the impact of the most relevant E3s (Table 1) and DUBs (Table 2) in the etiology of SCCs. We will discuss the signaling pathways regulated by these enzymes, emphasizing the genetic and molecular determinants underlying their deregulation in SCCs.

## 2. CRLs and SCC Etiology

The genomic profile of human SCCs has revealed that many CRL-related genes are subjected to cancer-associated genetic alterations, which include gene amplification, deletion and mutation [47]. Remarkably, the anti-tumorigenic action of the neddylation pathway inhibitor MLN4924 in various SCC subtypes clearly illustrates the critical role of CRLs activity in promoting and sustaining SCC pathogenesis [85,86,87]. In line with this, the expression signature of a subset of CRL-related genes defines HNSCC with unfavorable prognosis. In the following paragraphs, we will describe those CRLs for which genetic and biochemical studies have clearly established a relevant function in SCC etiology.

### 2.1. SCF^SKP2^: A Canonical Oncogene in SCCs

The S-phase kinase-interacting protein 2 (SKP2) is an F-box protein acting as a substrate receptor of the SCF complex SCF^SKP2^, a CRL1 crucially involved in the regulation of cell cycle progression, maintenance of genomic integrity and cancer development [58,59,88]. A number of evidences have clearly demonstrated that SKP2 acts as an oncogene in a variety of human tumors, including SCCs [60,61]. The initial observation of the oncogenic activity of SKP2 in SCC has been reported in 2002 [62]. Gstaiger et al. proved that SKP2 expression is positively correlated with the stages of SCC progression, as its expression increases during the transition from oral epithelial dysplasia to invasive SCC. More importantly, SKP2 cooperates with oncogenic mutant RAS to elicit a transformed phenotype in primary rat fibroblasts. Since then, a multitude of evidence has clearly established the pro-tumorigenic role of SKP2 in different SCCs, and many of its substrates underlying its oncogenic activity have been identified [89]. Among them, the CDK inhibitor p27 is likely to be the most relevant cancer-related SKP2 substrate [90,91,92]. In multiple tumors *p27* expression is mainly regulated at post-translational levels and its proteasome-dependent degradation represents the main pathway controlling its abundance [93,94]. Reduced p27 protein levels have been reported in oral precancerous lesions and carcinomas and are associated with higher proliferative index and upregulation of SKP2 [63]. During squamous carcinogenesis there is a marked expansion of the SKP2 positive cells and a corresponding reduction in p27-positive cells [64]. Notably, the inverse correlation between p27 and SKP2 proteins levels represents a negative prognostic factor in SCCs [65,66,67].

Although the SKP2-mediated degradation of p27 is likely to be critical for the oncogenic effects exerted by SKP2, it is plausible that additional SKP2 substrates might also play important functions during SCCs development. The long list of SKP2 substrates includes transcription factors (ELF4, SMAD4, TOL1, c-MYC and TOB1), chromatin modifiers (CDK9, MLL1 and MacroH2A1–CDK8) and growth factor signaling molecules (RASSF1 and MKP1) [58]. Interestingly, SMAD4 and MLL1 are mutated or downmodulated in some types of SCC, suggesting that these may function as tumor suppressors in SCCs [95,96]. In addition to trigger the proteasome-dependent degradation of its target proteins, SKP2 may also regulate non-proteolytic events. An example of such regulation is related to the activation of AKT signaling [97]. Activating mutation of PI3-kinase (PI3K) is one of the main oncogenic events driving squamous carcinogenesis and the PI3K-mediated activation of AKT signaling serves as a central node for the regulation of cell proliferation, survival and metabolism [98,99]. K63-linked ubiquitination of AKT is an important event necessary to activate AKT by regulating its membrane recruitment. It has been shown that in response to specific proliferative signals SKP2 is able to catalyze the non-proteolytic K63-linked ubiquitination of AKT and consequentially to enhance the oncogenic AKT-mediated pathways. Although this interesting non-proteolytic function of SKP2 has been characterized in breast carcinoma, it is reasonable to expect that the SKP2-AKT circuit may represent an alternative way to hyperactivate the AKT oncogenic signaling in those SCCs not harboring oncogenic mutation of PI3K and/or amplification of its upstream regulators (i.e., EGF-R).

The importance of SKP2 in SCCs pathogenesis is also enlightened by the fact that SCC-associated oncogenic events enhance Skp2 expression and activity in tumor cells. Activation of the PI3K-AKT pathway increases SKP2 expression, stability and activity in SCCs [100,101,102]. Another example concerns the role of the Hippo pathway in controlling SKP2 acetylation. The Hippo pathway is a signaling cascade crucially involved in controlling the organ size, stem cell self-renewal and tissue regeneration [103,104,105]. The core of the hippo pathway is a kinase cascade that induces the phosphorylation of the transcription factor YAP/TAZ, inhibiting its nuclear function [106]. It has been demonstrated that nuclear YAP/TAZ promotes SKP2 acetylation via AKT-p300 axis [107]. Acetylated SKP2 is exclusively localized in the cytosol, where it induces the degradation of the pro-apoptotic factors FOXO1/3. Remarkably, frequent amplification of YAP/TAZ and elevated nuclear staining of YAP/TAZ correlates with tumor aggressiveness, resistance to radiotherapy and poor outcome of SCC patients [105,108,109]. Furthermore, low expression of FOXO3 has been reported in HNSCCs [110]. Therefore, the YAP/TAZ-mediated acetylation of SKP2 and the subsequent cytosolic degradation of FOXO1 may represent an alternative route utilized by SCCs to enhance their survival.

Collectively, these data clearly establish the critical role exerted by SCF^SKP2^ in SCCs and represent the proof of concept that inhibition of SCF^SKP2^ activity may be exploited to dampen SCCs growth and survival.

### 2.2. Tumor Suppressor Activity of SCF^FBXW7^ in SCCs

The F-box protein FBXW7 is the substrate receptor of the SCF complex SCF^FBXW7^. According to the Cancer Genome Atlas (TCGA) data analysis, mutations of the *Fbxw7* gene are commonly observed in diverse SCCs [1,47]. Overall, SCCs have mutation frequencies in the range 3–14%, but there is substantial variation among SCC types. The highest mutation rates (approximately 14%) are found in vulval and CvSCCs, whereas HNSCCs as well as LSCCs have mutation frequencies in the range 5–20% and 4–6%, respectively. In addition to missense and nonsense mutations, loss of FBXW7 activity may be due to monoallelic and biallelic *Fbxw7* gene deletions, promoter hypermethylation or through the action of diverse FBXW7 targeting microRNAs (miRs) [68,69]. For instance, miR-223/miR-27a-3p and miR-24 are able to target FBXW7 and promote proliferation of ESCC and tongue SCC cells, respectively [70,111,112,113]. Elevated levels of miR-223 and miR-27a-3p are associated with poor prognosis of ESCC patients through the suppression of FBXW7. In line with these observations, decreased expression of FBXW7 is associated with a poorer prognosis of ESCC patients when compared to cases without a loss of FBXW7 expression [69,71,72].

The importance to restrict FBXW7 function during squamous carcinogenesis is also highlighted by the fact that p53, a positive regulator of FBXW7 expression [73], is commonly mutated in SCCs, suggesting that disruption of FBXW7 function can also be sustained by alterations in signaling pathways, such as mutation of p53, which allows tumor cells to gain hallmark properties.

The long list of the substrates targeted by FBXW7 include key cell cycle-regulatory molecules, such as c-MYC and CYCLIN E, anti-apoptotic proteins, such as MCL-1, and master regulator of squamous differentiation such as NOTCH1 and ΔNp63 [74]. In detail, c*-Myc* and *Notch1* have been identified among the target genes through which FBXW7 influences skin carcinogenesis by counteracting the proliferation-promoting effect of c-MYC and the tumor-suppressive effect of NOTCH1, respectively [75]. Consistent with this study, it has been shown that FBXW7 is required for NOTCH1 degradation during arsenite-mediated keratinocyte transformation [114,115].

It has been also found that FBXW7 targets ΔNp63 for polyubiquitination and subsequent degradation. ΔNp63 is highly expressed in the majority of SCC where it acts as an oncogene by regulating specific transcriptional programs aimed to sustain malignant cell proliferation, stemness and survival [116,117,118,119,120]. In a large panel of human cancer cell lines and patient biopsies, FBXW7 abundance was positively correlated with sensitivity to HDAC inhibitors (HDACi), which are known to downregulate ΔNp63 through FBXW7 to induce a tumor-suppressive program [121]. In a recent study, it has been shown that Sorting Nexin 5 (SNX5) is able to bind to FBXW7, to inhibit the degradation of NOTCH1, c-MYC and CYCLIN E1 [122]. Notably, high levels of SNX5 expression are indicative of an unfavorable prognosis in HNSCC patients.

As stated above, FBXW7 may also control the apoptotic machinery by regulating the degradation of the pro-survival factor MCL-1. In addition to modulating the apoptotic response by upregulating MCL-1, the FBXW7–MCL1 axis might have also a prognostic value for targeted approaches [123]. Indeed, He and colleagues reported that inactivation of FBXW7 determines a robust response to HDAC inhibitors (Vorinostat) in combination with Bcl-2-targeted compound (ABT-737) [124,125]. Therefore, FBXW7 inactivation might be utilized as a predictive marker for the therapeutic application of ABT-737/Vorinostat combination in SCCs.

Taken together, these data clearly demonstrate the crucial importance of FBXW7 tumor suppressor activity in SCCs development and support the notion that FBXW7 mutation might be exploited as a predictive marker to guide novel therapeutic treatments.

### 2.3. Keap1/Cul3/Rbx1 E3-Ubiquitin Ligase

The transcription factor Nrf2 codified by the gene *Nfe2l2* is a master regulator of the antioxidant cellular response [126]. Under physiological conditions, Nrf2 protein is recognized by Keap1, the receptor subunit of the CRL3 complex Keap1/Cul3/Rbx1, which triggers its degradation [127]. In response to increased levels of oxidative stress, a conformational change of Keap1 abolishes Keap1-Nrf2 interaction, leading to Nrf2 stabilization and activation of the Nrf2 responsive genes involved in antioxidant defense, glucose and lipid metabolism, apoptosis, autophagy, metastasis and chemoresistance [128]. In human tumors the outcome of Nrf2 activity is strictly context-dependent and Nrf2 has been traditionally considered as a tumor suppressor for its cytoprotective functions against exogenous and endogenous insults [129,130]. On the other hand, many tumor cells are characterized by elevated Nrf2 activity and under oncogenic stimuli such as those induced by KRAS, BRAF or PI3K-AKT signaling Nrf2 acts as an oncogene promoting proliferation and tumorigenesis [131,132]. The cancer genomic profiling along with global methylation and transcriptome studies have clearly identified distinct genetic and molecular mechanisms responsible for Nrf2 activation in various types of cancer, including SCCs. In SCCs the Keap1/Cul3/Rbx1 E3 is compromised by multiple mechanisms including mutation of *Cul3* and *Keap1* genes and *Keap1* promoter hypermethylation [47,76,133]. The majority of tumor-associated *Keap1* mutations either abrogates its binding to Nrf2 or decreases its associated E3 ligase activity. The characterization of animal models lacking the *Keap1* gene has further validated the relevance of Keap1 during squamous carcinogenesis. Indeed, the genetic deletion of Keap1 together with *Tp53* loss in airway basal stem cells generates tumors recapitulating histologic and molecular features of human LSCCs [77].

Mutations of *Nfe2l2* has been reported in all subtypes of SCCs, including esophagus (11.4%), skin (6.3%), lung (8.0%) and larynx (13.0%) SCCs [12,13,14,15]. Of note, *Nfe2l2* mutations are located within or near motifs (DLG and ETGE) that are important for Nrf2 and KEAP1 interaction and generally are mutually exclusive with *KEAP1* mutations [134]. Notably, the keratinocytes-specific expression of Nrf2 mutant lacking the Keap1-binding domain promotes HPV8-induced skin papilloma formation in mice [78]. If we collectively consider the mutations of the Cul3-KEAP1-Nrf2 axis, 64% and 34% of HNSCC and lung SCC patients have alteration of this pathway, respectively, and survival for patients harboring disruption to any component (KEAP1, Cul3 and/or RBX1) show poorer median survival compared with patients without alterations [71,79,80,81]. The mutation rates of *Cul3, Keap1* and *Nfe2l2* genes are significantly higher in HPV negative tumors respect to the positive one [12]. By mapping the protein interaction network of HPV proteins a recent report found that the HPV E1 protein binds to and inactivates KEAP1, thereby leading to enhanced expression of NRF2 target genes [135].

Collectively, these data clearly indicate that SCCs have evolved different mechanisms aimed to sustain Nrf2 mediated signaling, that ultimately enhance the survival of SCC cells facing excessive oxidative stress, chemotherapeutic agents or radiotherapy.

## 3. The APC/C Complex

SCCs are characterized by complex genomic alterations including aneuploidy, chromosomes translocations and deletions [136]. Mechanistically, aneuploidy is mainly due to aberrant mitotic events, and aberrant mitotic figures such as anaphase bridges and multipolar mitotic divisions have been documented in HNSCC primary tumors and oral cancer cell lines [137,138,139]. Remarkably, the chromosomal instability (CIN) status is associated with an unfavorable prognosis of oral cancer patients [140].

The APC/C (anaphase-promoting complex/cyclosome) complex is likely the most relevant E3 controlling key events of mitotic progression [141]. The activity of the APC/C is finely regulated and is dependent on the binding of two adaptors, Cdc20 and Cdh1/FZR1 that function as substrate recruitment factors in specific phases of mitosis. The activity of Cdc20 is strictly regulated by a highly conserved surveillance mechanism, the spindle assembly checkpoint (SAC) that ensures the accurate chromosome distribution between the two daughter cells, only in the presence of attached and aligned chromosomes [142].

Several reports have suggested that the activity of APC/C-Cdc20 is dysregulated in SCCs. An initial observation indicated that Cdc20 levels are increased in HNSCC cell lines and primary HNSCC tissues [82]. The increased expression of Cdc20 is associated with the defective response to the SAC, and is correlated with abnormal chromosome number and multinucleation in HNSCC cells. Furthermore, exogenous overexpression of Cdc20 in a chromosomally stable HNSCC cell line determines an impairment of SAC function and the CIN phenotype [82]. These initial data have been subsequently confirmed in other types of SCCs [83,84]. A recent systematic analysis of UPS machinery genomic alterations has indeed revealed that amplification/overexpression of diverse APC/C components such as Cdc20, Apc11, Cdc27 Apc1 and Apc3 occurs in HNSCCs [47]. Remarkably, in HNSCC patients this APC/C signature is predictive of poor prognosis [47]. Consistently, Cdc20 overexpression in oral SCC histological samples has been linked to a poor prognosis, suggesting Cdc20 as a novel independent prognostic factor, as well as a molecular marker to categorize high-risk oral cancers subgroups [143].

The role of Cdh1 in SCCs development is more complex, likely due to the broad spectrum of Cdh1 substrates involved in many processes beyond cell cycle progression [144]. As a general concept, Cdh1 is described as a haploinsufficient tumor suppressor gene and Cdh1 heterozygosity results in the development of epithelial tumors [145]. Down-regulation of Cdh1 is observed in several cancers and many Cdh1 substrates, such as M/S-phase cyclins and DNA replication factors are frequently overexpressed in tumors [144]. An interesting report showed that APC/C-Cdh1 induces the ubiquitin-proteasome dependent degradation of ΔNp63 [146]. Rokudai et al. found that APC/C-Cdh1 regulates ΔNp63 protein levels both in primary keratinocytes and in SCC cell lines. A mutant ΔNp63 refractory to the APC/C-Cdh1 regulation displays increased oncogenic activity. Interestingly, the APC/C-Cdh1-mediated degradation of ΔNp63 is counteracted by the activity of the syntaxin-binding protein 4 (Stxbp4), which is frequently overexpressed in LSCC [147]. In LSCC, Stxbp4 levels are significantly correlated with ΔNp63 accumulation and with unfavorable prognosis [148]. These data suggest that in SCC the APC/C-Cdh1 complex may exert a tumor suppressive function by, at least in part, targeting ΔNp63. 

## 4. MDM2 and E6AP Restrain p53 in SCCs

Although inactivating mutations of the *Tp53* gene are frequently observed in the majority of SCCs, two cellular signaling pathways involving the MDM2 and E6AP E3s contribute to dampen p53 tumor suppressor activity in p53 proficient SCCs. MDM2 (mouse double minute 2) is a RING-type E3 able to triggering the proteasome-dependent degradation of p53 in normal conditions. Upon a variety of stress signals p53 undergoes various post-translational modifications that allow its escape from MDM2 binding and modification [149,150]. HPV infection also exploits the ubiquitin–proteasome system to restrain p53 activity. In particular, high risk HPV E6 protein recruits the cellular HECT-domain ubiquitin ligase E6AP and redirects its substrate specificity towards p53 [44]. MDM2 is often amplified/overexpressed in human tumors, especially in soft tissue cancers, where its elevated levels correlate with both tumor grade and poor prognosis [149]. In SCCs amplification/overexpression of MDM2 is observed at low frequency, with HNSCCs displaying the highest rate of gene amplification (approximately 4% of the cases, according to the TCGA). On the other hand, SCCs frequently harbor deletions of the *Cdkn2a* genetic locus that may cause the loss of the p14/ARF protein, a negative regulator of MDM2 [10,151]. MDM2 contribution to SCC tumorigenesis seems to be particularly relevant in ESCCs patients where MDM2-positive lesions show a shorter overall survival [48,49]. In addition, a promoter polymorphism (SNP309) that enhances the binding affinity of the Sp1 transcription factor and results in elevated levels of MDM2 protein is a risk factor to ESCC and is associated with poorly differentiated and advanced forms of this type of cancer [50].

Interestingly, in SCCs MDM2 amplification/overexpression can also occur in p53 wild type tumors, suggesting p53-independent function. p53-independent MDM2 functions include the ubiquitination and degradation of cell-cycle regulators, such as Rb, p21 and FoxO3A [51,52]. With the exception of ESCCs, Rb mutations are quite rare but its activity is suppressed by different mechanisms such as mutation or deletion of the CDK inhibitor CDKN2A, expression of HPV E7 proteins and MDM2 overexpression itself [10].

MDM2 has been also shown to ubiquitinate and target for degradation E-cadherin [53]. Interestingly, low protein expression of E-cadherin in oral SCCs correlates with recurrence, poor cellular differentiation, metastatization and shorter overall survival [152,153]. Moreover, E-cadherin expression is positively correlated with the stages of oral SCC progression, as its expression increases during the transition from dysplasia to oral cancer [154,155].

## 5. Additional E3s Involved in SCCs

TRIM32 is a member of the tripartite motif protein family characterized by a tripartite motif that includes the RING domain B1 and/or B2 boxes, and the coiled-coil domain [156]. Similarly to other members of this family, TRIM32 is involved in cancer development and its pro-oncogenic action has been described in different tumor contexts, including SCCs [54,55,56]. TRIM32 levels are upregulated during UVB-induced squamous carcinogenesis and in a fraction of chemically induced mouse papilloma [54]. Human HNSCC samples also display an elevated level of TRIM32 [54]. Although different TRIM32 substrates underlying its pro-oncogenic function have been identified, a recent report uncovered a novel oncogenic route exploited by TRIM32 in SCCs [57]. Luo et al reported that TRIM32 is able to target the SWI/SNF complex subunit ARID1A, a potential tumor suppressor. According to the TCGA, the *Arid1a* gene is mutated at relatively low frequency in SCCs [12,13,14]. Nevertheless, SCCs are characterized by low ARID1A protein expression, indicating that the TRIM32-dependent degradation of ARID1A may represent a relevant mechanism contributing to underexpress this epigenetic factor in SCC. Notably, TRIM32 action is counteracted by the DUB USP11, which promotes the stabilization of ARID1A protein [57]. Consistently, SCC samples show a negative correlation between TRIM32 and ARID1A expression and a positive correlation between USP11 and ARID1A expression. Taken together this data suggest a model in which the relative expression levels of TRIM32 and USP11 may finely determine ARID1A protein levels, thereby affecting SCCs development.

Another E3 ligase potentially implicated in SCC biogenesis is the HECT-type E3 ligase WWP1. Similarly to what was observed in other tumor contexts, WWP1 likely acts as an oncogene also in SCCs and elevated levels of WWP1 has been reported in HNSCC tissues and in several oral cancer cell lines [45]. Consistently, WWP1 depletion in cutaneous SCCs and HNSCC cells decreases tumor cell proliferation [46]. Although these data confirm the general view of WWP1 as a positive regulator of tumorigenesis, further in vivo evidence is required to assess its contribution during squamous carcinogenesis as well as to uncover the molecular mechanisms underlying its oncogenic function in SCCs.

## 6. DUBs

A plethora of genetic and biochemical studies have clearly established the critical role of DUBs in controlling physiological and pathological events including cancer development [157]. Furthermore, genomic sequencing studies have revealed that DUBs are targets of many genomic insults such as deletions, mutations and copy number variations (CSN) [47]. In the following paragraphs, we will present an overview of the DUBs whose activity or expression have been linked with SCC pathogenesis (see Table 2).

### 6.1. USP22

USP22 is a conserved ubiquitin hydrolase whose predominant function is related to the removal of the monoubiquitin moiety from histone H2B [158,175]. USP22 deubiquitination activity towards histone H2B is strictly dependent on its association within the nuclear multiprotein Spt-Ada-Gcn5 acetyltransferase (SAGA) complex, mainly involved in transcription regulation [176]. Many transcription factors, such as c-Myc and p53, implicated in tumor development require the association with the SAGA complex in order to activate the expression of target genes [175]. Based on these considerations, it is not surprising that USP22 possesses oncogenic activity and that elevated expression of USP22 characterizes several human tumors, including SCCs. The initial observation of the potential oncogenic role of USP22 in SCC emerged in 2012, when a study analyzed USP22 expression in ESCC tissues by immunohistochemistry (IHC) [159]. High expression of USP22 protein has been detected in the majority of ESCC tissues and it is significantly associated with lymph node metastasis, pathologic stage and tumor relapse. Consistently, ESCCs patients with high expression of USP22 protein have an unfavorable prognosis. The oncogenic ability of USP22 has been sub-sequentially confirmed in HNSCCs [160,161]. In detail, USP22 expression is increased in parallel with the progression of oral carcinogenesis, from non-cancerous mucosa to primary carcinoma and from carcinomas to lymph node metastasis. Remarkably, oral SCC patients with positive USP22 expression have significantly poorer outcome compared to patients with negative expression. 

As stated above, the predominant role of USP22 is to catalyze the removal of monoubiquitin moiety from H2B and it is likely that this function is fundamental for USP22 oncogenic activity. However, the dynamic regulation of mono-Ub-H2B depends not only on the activity of USP22 but also on the E3 ubiquitin ligase complex RNF20/RNF40, which catalyzes the monoubiquitylation of H2B [177]. Interestingly, the HPV protein L2 is able to bind the RNF20/RNF40 complex and phenocopy the invasive phenotype promoted by inactivating mutations of RNF20/RNF40 in HPV negative HNSCC [135]. This suggests that the regulation of the abundance of the mono-ub-H2B is an important oncogenic outcome of USP22-RNF20/40 activity in SCCs. However, USP22 is also able to deubiquitinate other targets such as the deacetylase SIRT1 [178], which might contribute to the tumorigenic capability of USP22. Therefore, more comprehensive studies are required to clarify the impact of USP22/RNF20-RNF40 circuit as well as the contribution of the growing number of USP22 substrates on squamous carcinogenesis.

### 6.2. USP48 and BRCC3

The genomic profiling of human tumors has revealed that the *USP48* locus is commonly depleted in the majority of SCCs, such LSCCs, ESCCs and HNSCCs [47]. USP48 is a DUB that counteracts the E3 ligase activity of the complex formed by the breast cancer type 1 susceptibility protein (BRCA1) and its obligatory partner BRCA1-associated RING domain protein 1 (BARD1) [179,180,181]. BRCA1-BARD1 complex mediates the ubiquitination of histone H2A at specific lysine residues, an important event for reposition p53BP1 and allow end resection, a crucial early step in the repair of double strand breaks (DSBs) by homologous recombination (HR) [162,182,183]. By limiting the extent of H2A ubiquitination, USP48 affects the repositioning of p53BP1 at DNA damaged sites, thus limiting resection length [180,184]. USP48 depleted cells show extensive resection, which can result in the use of a sub-pathway of HR repair known as single-strand annealing (SSA) [180]. Conversely, USP48 overexpression phenocopies aspects of BRCA1 loss. In both cases these events could be mutagenic and favor genome instability. Alternatively, since USP48 depleted cells show increased resistance to camptothecin [180], USP48 underexpression might impact the chemotherapeutic response of SCC cells. It is worth noting that somatic mutations of the *BRCA1* gene appear to be very rare in SCCs. Therefore, USP48 inhibition might represent an alternative pathway to restrain BRCA1-mediated activity in SCCs.

Another BRCA1-related DUB possibly involved in SCCs pathogenesis is BRCC36. BRCC36 (the human orthologous of the mouse BRCC3 gene) is a JAMM domain-containing Zn2+ metalloprotease taking part in two multiprotein complexes: BRCA1-A and BRISC. The BRCA1-A complex finely regulates the activity of BRCA1 in the HR pathway, since it is able to sequester BRCA1 away from DNA break sites [185,186], thus suppressing resection and ultimately limiting HR ([187]. The BRCA1-A assembly is regulated by RNF8/RNF168-mediated ubiquitination of H2AX [188]. BRCC36 counteracts the action RNF8/RNF168 at DNA damaged sites and specifically cleaves K63-linked ubiquitinated histones, [163,189]. Elevated levels of BRCC3 have been reported in nasopharyngeal carcinoma and high BRCC3 expression is associated with radio-resistance and poor survival of nasopharyngeal carcinoma patients [163]. In agreement with these results, BRCC36 gene amplification has been found in HNSCCs, ESCCs and CvSCCs [47].

In addition to being part of the BRCA-A1 complex, BRCC36 is also a subunit of the cytosolic complex BRISC, an important regulator of the immune signaling. In detail, BRCC36 controls the K63-ubiquitin levels of a wide range of immune-related substrates including ATF4, THAP5 and the inflammasome component NLRP3 [190,191]. BRCC36 is able to de-ubiquitinate NLRP3 favoring the activation of the inflammasome and the subsequent synthesis of pro-inflammatory factors such as interleukin 1B [191]. Remarkably, NLRP3 activity has been linked to the pathogenesis of different tumors, including SCCs [192]. Elevated levels of NLRP3 have been found in ESCCs, LSCCs and HNSCCs and high expression of NLRP3 is a negative prognostic factor in patients with LSCC [193]. Furthermore, biochemical studies demonstrated that dysregulation of NLRP3 might influence the oncogenic features of SCC cell lines [194]. 

Collectively, these data indicate that BRCC36 might impact two processes commonly deregulated during SCC development: DNA repair and inflammatory response, representing thus a potential therapeutically actionable target.

### 6.3. USP28

USP28, one of the most studied DUBs, controls key events of tumor progression [164]. One central function of USP28 is related to its ability to counteract the tumor suppressor activity of SCF^FBXW7^ and promote the stabilization of several cell cycle-related proteins, such as c-Myc and cyclin E. As a result, USP28 activity has been linked to enhanced proliferation, carcinogenesis and metastasis [164]. A recent report identified another USP28-mediated pathway potentially contributing to squamous carcinogenesis [165]. Prieto-Garcia and colleagues reported that USP28 is able to bind to and promote the deubiquitination of ΔNp63 (see also paragraph 2.2). Genetic or chemical inhibition of USP28 decreases SCC proliferation and impacts the expression of epithelial target genes in diverse SCC cells, thus phenocopying the effect of ΔNp63 silencing. These results have been also confirmed in a mouse model of LSCCs, as genetic loss of USP28 hinders SCC growth. Remarkably, human LSCC tissues display a strong correlation between USP28 and ΔNp63 expression, and patients with elevated levels of USP28 have an unfavorable prognosis.

In addition to maintaining the epithelial identity and promoting tumor growth through ΔNp63 protein stabilization, USP28 oncogenic activity might also be related to other signaling pathways. Indeed, USP28 controls the stability of factors and mediators of the ATR/ATM signaling such as Claspin and p53BP1, which are important to finely regulate DNA replication, S-phase checkpoint and G2/M arrest in response to DSBs [195,196]. Notably, Claspin is upregulated in many different cancers, including CESCCs and its increased expression is associated with a bad prognosis [197]. One possibility is that USP28 mediated stabilization of Claspin may confer a proliferative advantage to SCC cells by ensuring a proper replication fork progression in cells experiencing chronic replicative stress. Taken together, these results indicate that the protease USP28 may regulate distinct oncogenic routes, each of them likely contributing to maintain the tumor phenotype of SCCs.

### 6.4. USP9x

USP9x (also known as FAM) is a highly conserved USP-type DUB, whose activity has been implicated in regulating factors involved in various signaling pathways, such protein trafficking, cell death, stemness and cell polarity [198]. In addition to regulating physiological processes, USP9x activity has been also linked to pathological events, including cancer. The role of USP9x in cancer development is quite complex as it can act as both a tumor suppressor and an oncogene. This dichotomy is probably related to the broad range of USP9x substrates, which include survival proteins (β-catenin and the BCL-2 family member MCL1), pro-apoptotic proteins (ASK-1 and DLK kinase), TGF-β signaling effectors (SMAD4 and SMURF1) and mTOR pathway factors [198,199,200,201]. 

Similarly to other cancers, contrasting evidence on the role of USP9x in SCCs has been reported. Indeed, while IHC studies on ESCC tissues revealed that USP9x is expressed at high levels in this type of SCC and it correlates with lymph node metastasis and poorer survival rate [166,167], other reports indicate that down-modulation of USP9x rather than its upregulation characterizes oral SCC [168,169]. The genome profiling of 60 gingival SCCs revealed that approximately 20% of oral SCC tissues harbors copy number loss and truncating mutations in *USP9x* locus consistent with a potential role as a tumor suppressor [169]. Consistently, USP9x is also mutated in a panel of oral SCC cell lines [170]. In vivo studies on cancer mouse model are also indicative for a tumor suppressor role of USP9x in SCC. The Cre-mediated genetic inactivation of *USP9x* enhances the KrasG12D-driven tumorigenesis in pancreatic tissues and accelerates the development of aggressive oral papillomas [171]. These studies clearly suggest that USP9x activity might be relevant for squamous carcinogenesis. However, additional investigations are required to clarify the molecular pathways underlying its function in SCCs. 

### 6.5. OTUD3 and USP7: a Two-Way Mode to Dampen PTEN in SCCs?

Another interesting DUB in the context of SCC carcinogenesis is the OTU domain-containing protein 3 (OTUD3). Members of the OTU family controls important cell signaling pathways, such as NF-κB signaling and the DNA damage response, and are involved in several processes such as innate immune signaling and angiogenesis [202]. The genomic profiling of human cancers revealed that the *Otud3* locus is targeted by gene deletion hits that may impair its function in multiple cancers, including HNSCCs, LSCCs and CESCCs [47]. The molecular bases of the putative tumor suppressor role of OTUD3 in SCCs have not been fully clarified. One possibility is that impairment of the OTUD3 function in SCCs, similarly to what was observed in breast carcinoma, may destabilize the tumor suppressor PTEN, thereby activating the AKT-mediated oncogenic signaling [203]. Notably, USP7 (also known as HAUSP, herpesvirus-associated ubiquitin-specific protease) has been also implicated in PTEN deubiquitilation process [204]. USP7 is able to remove the monoubiquitin moiety from PTEN, causing PTEN’s exclusion from the nucleus, thereby inhibiting the nuclear-associated tumor suppressor function of PTEN [204]. 

USP7 overexpression has been described in HNSCCs, ESCCs and LSCCs [172,173,174]. In ESCC cells downmodulation of USP7 expression decreases tumor proliferation, impairs cell migration and invasion and induces the expression of pro-apoptotic genes (e.g., NOXA) [172]. One major function of USP7 is to regulate the crosstalk between the tumor suppressor p53 and its cognate E3 ligase MDM2 [205,206]. However, USP7 may have p53-independent functions, which may be relevant in the context of SCCs since in these tumors p53 mutations are the most common genetic alterations. Therefore, the USP7-mediated regulation of PTEN activity might be an important outcome of the USP7 oncogenic activity in SCCs.

Collectively, two SCCs-associated events (USP7 overexpression and OTUD3 deletion) may cooperate to dampen PTEN tumor suppressor activity. Of course, other USP7 substrates, such as transcription factors, epigenetic modulators and cell death-related proteins, might be relevant for the oncogenic activity of USP7 in SCCs. Furthermore, the limited number of OTUD3 substrates identified so far and the lack of in vivo studies do not allow us to draw unequivocal conclusions.

## 7. Concluding Remarks and Perspective

**Figure 2:**
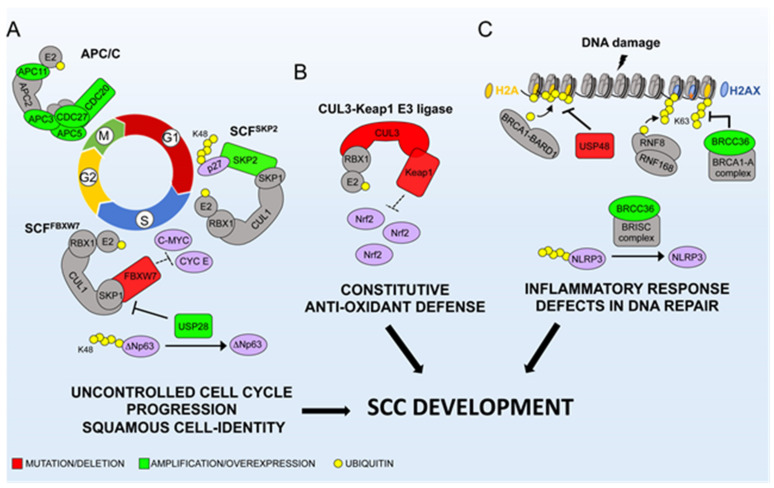
Mechanistic model describing the biological processes underlying the main molecular and genetic alterations of the ubiquitin system in SCCs. The indicated ubiquitin system-related factors are involved in cell cycle progression (**A**), anti-oxidant defense (**B**) and DNA damage checkpoint/inflammatory response (**C**). See text for details.

SCCs are highly malignant cancers with limited therapeutic options. In this review, we described how alterations of E3s and DUBs impact on important cellular signaling pathways, thereby fostering the initiation and progression of SCCs. In a simplistic way, three major processes are deregulated by the ubiquitin system in SCC: cell cycle progression, DNA damage/DNA repair and oxidative stress response (Figure 2). Distinct genetic and molecular events cooperate to boost the oncogenic activity of SCF^Skp2^, to dampen the tumor-suppressor function of SCF^FBXW7^ and to deregulate the activity of APC/C-Cdc20 complex during mitosis (Figure 2A). These events ultimately promote the uncontrolled cell cycle progression and favor aneuploidy, two cellular phenomena commonly observed in the majority of SCCs. Activation of the Nrf2-mediated pro-tumorigenic pathway by inactivating mutations of Cul3-KEAP1 ligase components or of the *Nfe2l2* gene is also frequently observed in many SCCs (Figure 2B). The end product of these genetic events is the constitutive activation of Nrf2, which may favor SCCs progression by inducing pro-tumorigenic pathways and/or dampening excessive oxidative stresses generated by endogenous or exogenous insults. It is interesting to note that the etiology of SCC of diverse tissues is tightly linked with the exposure to several environmental insults, such as tobacco use, alcohol consumption and environmental toxins, which may ultimately induce excessive oxidative stresses. Therefore, during SCCs progression tumor cells could have evolved distinct mechanisms to enhance the activity of Nrf2, thereby favoring SCC growth and dissemination.

The third signaling pathway highly deregulated in SCCs is related to the activation of DNA repair/DNA damage response. Genetic and molecular events affecting components of BRCA1-BARD1-USP48 and BRCC36 complexes might enhance genomic instability and increase the resistance to chemotherapy drugs (Figure 2C).

This view, although oversimplified, may represent the starting point to tailor specific therapeutic intervention, which combines specific inhibitors of E3 ligases and DUBs. For instance, a potential combination therapeutic strategy might involve the inhibition of the Skp2–Skp1 interaction by compound #25 [207] and a putative inhibitor of USP28. USP28 inhibition would have three major biological outcomes: enhancing the degradation of key cell cycle factors, such as c-Myc and cyclin E, destabilizing ΔNp63 and deregulating the DNA damage response. As a result, the combined action of Skp2 and USP28 inhibitors could result in a strong inhibition of tumor growth and enhanced response to conventional anti-neoplastic drugs. These considerations should boost our effort to identify and characterize USP28 specific inhibitors, which may be beneficial for SCCs treatment. Along the same line, inhibition of NRF2 activity might also be pursued to dampen the cellular defense against oxidative stress. Accordingly, NRF2 inhibitory compounds have promising therapeutic potential for diminishing survival of LSCC [208].

In conclusion, aberrant activity of the ubiquitin system certainly contributes to the initiation and progression of SCCs, and key factors of this intricate network might represent an attractive, albeit complex, molecular target for SCC treatment. However, an improved understanding of ubiquitin networks in SCC is still necessary and the identification and characterization of novel substrates of E3s or DUBs will enable to unveil novel therapeutically actionable signaling pathways involved in SCC pathogenesis.

## Figures and Tables

**Table 1:** Deregulation of the E3s in squamous cell carcinomas (SCCs).

E3 Ligase	Function	Cancer-Associated Substrates	Alterations in SSC Cells	Clinical Correlations	References
E6-AP	HECT-type E3	p53, PML	In HPV-positive SCCs, E6-AP associates with E6 and acquires substrate specificity towards p53 (oncogene)	It contributes to HPV-driven carcinogenesis	[44]
WWP1	HECT-type E3	p53, ΔNp63, p27^Kip1^, Notch, Smad2, Smad4	Overexpression in HNSCC patients (oncogene)	In CSCC patients, high expression is associated with lymph node metastasis.	[45,46]
MDM2	RING-type E3	p53, Rb, p21, FoxO3A, E-cadherin	Amplification, overexpression, hyperactivation as a result of p14/ARF loss (oncogene)	Correlation with SCC progression, adverse prognosis and chemoresistance	[47,48,49,50,51,52,53]
TRIM32	RING-type E3	ARID1A	Overexpression in HNSCC (oncogene)	N.D.	[54,55,56,57]
SKP2	F-box protein, substrate receptor of the CRL1 E3 (SCF complex)	p27, AKT, Smad4, Tob, c-Myc, cdk9, Mll1, Rassf1, Mkp1	Overexpression (oncogene)	Correlation with SCC progression, lymph node metastasis and radioresistance	[58,59,60,61,62,63,64,65,66,67]
FBXW7	F-box protein, substrate receptor of the CRL1 E3 (SCF complex)	c-Myc, Notch1, Cyclin E, MCL-1, ΔNp63	Missense and nonsense mutations, gene deletion, promoter hypermethylation, miRNA (tumor suppressor)	Correlation with adverse prognosis of ESCC patients and reactivity to chemoradiation therapy	[11,12,13,14,15,68,69,70,71,72,73,74,75]
KEAP1	Substrate receptor of the CRL3 E3	Nrf2	Mutations, promoter hypermethylation, inactivation by HPV (oncogene)	Correlation with adverse prognosis in HNSCC. In LSCC mutations are associated with poor response to adjuvant therapy, and poorer overall survival.	[11,12,13,14,15,47,71,76,77,78,79,80,81]
CUL3	Cullin subunit of the CRL3 E3	Nrf2	Mutations (oncogene)	Correlation with adverse prognosis in HNSCC and lung SCC patients	[47]
CDC20	Substrate adaptor of the APC/C E3 complex (early mitosis)	Cyclin A, Cyclin B1, Securin	Amplification, overexpression (oncogene)	An APC/C signature is predictive of poor prognosis in HNSCC patients	[47,82,83,84]

N.D.: not determined.

**Table 2:** Deregulation of the DUBs in SCCs.

DUB	Class	Cancer-Associated Substrates	Alterations in SSC Cells	Clinical Correlations	References
USP22	USP	H2B, SIRT1, FBP1	Overexpression (oncogene)	Positive correlation with SCC progression and lymph node metastasis, poor prognosis	[158,159,160,161]
USP48	USP	H2A	Deletion (tumor suppressor)	Genomic instability	[47]
BRCC36 (BRCA1or BRISC complex)	JAMM	H2A, γH2AX, NLRP3, ATF,	Overexpression (oncogene)	Radioresistance, poor prognosis	[47,162,163]
USP28	USP	ΔNp63, Claspin, 53BP1, FBW7	Overexpression (oncogene)	Poor prognosis	[164,165]
USP9x	USP	β-catenin, MCL1, ASK-1, DLK, SMAD4, SMURF1	Deletion, mutation (tumor suppressor) in oral cancer. In ESCC high expression	In ESCC high expression correlates with lymph node metastasis and poorer survival rate	[166,167,168,169,170,171]
OTUD3	OTU	PTEN	Deletion (tumor suppressor)	N.D.	[47]
USP7	USP	p53, MDM2, PTEN	Overexpression (oncogene)	In LSCC correlation with a more advanced tumor stage, lymph node metastasis and shorter overall survival	[172,173,174]

N.D.: not determined.

## Abbreviations

SCCs	squamous cell carcinomas
HNSCCs	squamous cell carcinomas of head and neck
LSCCs	lung squamous cell carcinomas
ESCCs	esophageal squamous cell carcinomas
CvSCCs	cervical squamous cell carcinomas
CSCCs	cutaneous squamous cell carcinomas
E3s	E3 ubiquitin ligases
DUBs	deubiquitinating enzymes
CDK	cyclin-dependent kinase
EGF-R	epidermal growth factor receptor
HPV	human papilloma viruses
PI3K	PI3kinase: phosphatidylinositol 3-kinase-Protein Kinase B
APC/C	anaphase-promoting complex/cyclosome
HDAC1	histone deacetylases 1
HDAC2	histone deacetylases 2
FGFR2	Fibroblast Growth Factor Receptor 2
CSN	copy number variations
VEGF	Vascular endothelial growth factor
DSB	DNA double-strand break
HR	homologous recombination
NHEJ	non-homologous end joining
BRCA1	breast cancer type 1 susceptibility protein
BARD1	BRCA1-associated RING domain protein 1
TGFβ	transforming growth factor beta
NF-κB	nuclear factor kappa-light-chain-enhancer of activated B cells
